# SHP2 Positively Regulates TGFβ1-induced Epithelial-Mesenchymal Transition Modulated by Its Novel Interacting Protein Hook1*[Fn FN2]

**DOI:** 10.1074/jbc.M113.546077

**Published:** 2014-10-20

**Authors:** Shuomin Li, Linrun Wang, Qingwei Zhao, Yu Liu, Lingjuan He, Qinqin Xu, Xu Sun, Li Teng, Hongqiang Cheng, Yuehai Ke

**Affiliations:** From the ‡ Department of Pathology and Pathophysiology, Program in Molecular Cell Biology, Zhejiang University School of Medicine, Hangzhou 310058, China,; § The First Affiliated Hospital, College of Medicine, Zhejiang University, Hangzhou 310003, China, and; ¶ Collaborative Innovation Center for Diagnosis and Treatment of Infectious Diseases, Hangzhou, Zhejiang 310003, China

**Keywords:** Epithelial to Mesenchymal Transition, Epithelial-Mesenchymal Transition (EMT), Metastasis, Protein Phosphatase, Protein-Protein Interaction, Hook1, Mesenchymal, Protein-tyrosine Phosphatase, shp2

## Abstract

The epithelial-mesenchymal transition (EMT) is an essential process for embryogenesis. It also plays a critical role in the initiation of tumor metastasis. Src homology 2 (SH2)-domain containing protein-tyrosine phosphatase-2 (SHP2) is a ubiquitously expressed protein-tyrosine phosphatase and is mutated in many tumors. However, its functional role in tumor metastasis remains largely unknown. We found that TGFβ1-induced EMT in lung epithelial A549 cells was partially blocked when SHP2 was decreased by transfected siRNA. The constitutively active form (E76V) promoted EMT while the phosphatase-dead mutation (C459S) and the SHP2 inhibitor PHPS1 blocked EMT, which further demonstrated that the phosphatase activity of SHP2 was required for promoting TGFβ1-induced EMT. Using the protein-tyrosine phosphatase domain of SHP2 as bait, we identified a novel SHP2-interacting protein Hook1. Hook1 was down-regulated during EMT in A549 cells. Overexpression of Hook1 inhibited EMT while knockdown of Hook1 promoted EMT. Moreover, both the protein-tyrosine phosphatase domain and N-terminal SH2 domain of SHP2 directly interacted with Hook1. Down-regulation of Hook1 increased SHP2 activity. These results suggested that Hook1 was an endogenous negative regulator of SHP2 phosphatase activity. Our data showed that the protein-tyrosine phosphatase SHP2 was involved in the process of EMT and Hook1 repressed EMT by regulating the activation of SHP2. SHP2-Hook1 complex may play important roles in tumor metastases by regulating EMT in cancer cells.

## Teaser

**Background:** SHP2 regulates cancer development and progression.

**Results:** SHP2 promotes TGFβ1-induced EMT requiring its phosphatase activity. PTP and N-SH2 domains of SHP2 interact with Hook1, which negatively regulates TGFβ1-induced EMT.

**Conclusion:** SHP2-Hook1 complex regulates TGFβ1-induced EMT.

**Significance:** SHP2 plays important roles in tumor metastases due to promoting EMT in cancer cells.

## Introduction

Metastasis is the leading cause of mortality in human cancer (1). There are several stages during metastasis, including local invasion, hematogenous spread, and colonization. The epithelial-mesenchymal transition (EMT)^3^ participates in the initiation stage of metastasis, and endows the cancer cells with migratory and invasive properties (2). EMT has also been found to participate in the processes of development, fibrosis, and wound-healing (3).

EMT is a form of cell differentiation. Previous reports have shown that the Src homology 2 (SH2)-domain containing protein-tyrosine phosphatase-2 (SHP2) plays important roles in cell differentiation, indicating that it might participate in the regulation of EMT (4–6). SHP2, a member of the non-receptor protein-tyrosine phosphatase family, participates in many signal transduction and activation of the Ras-ERK signaling pathway, mostly beginning by EGF binding to the EGF receptor (7, 8). This also suggests that SHP2 may play regulatory roles in the EMT process (9, 10). SHP2 also plays important roles in development, cancer, inflammation, transcription regulation, and cell migration (11–15).

SHP2 contains two SH2 domains, N-SH2 and C-SH2 domain, at its N terminus. In the basal state, the N-SH2 domain binds to the PTP domain and blocks the active site. Thus, the activity of SHP2 is inhibited. With stimulation, the N-terminal SH2 domain binds to a phosphorylated tyrosine residue, detaches from the PTP domain, and SHP2 is activated (16, 17). Germline mutations in SHP2 encoding gene *PTPN11* are associated with Noonan syndrome, LEOPARD syndrome, and metachondromatosis (18–21). Somatic activated SHP2 mutations have also been detected in acute myeloid leukemia, neuroblastoma, melanoma, breast cancer, lung cancer, and colorectal cancer (22, 23). These data suggest that *PTPN11* functions as a proto-oncogene. However, it has been recently reported that SHP2 can act as either a tumor promoter or suppressor (24–26). Depending on the tissues and disease stages, SHP2 plays different roles in different tumors. The exact role of SHP2 in cancer, especially metastasis, is not clear.

It has been reported that several growth factor and cytokine factor signaling pathways (TGFβ1, FGF, EGF, HGF, Wnt/β-catenin, and Notch) participate in EMT (27). In A549 cells (human lung adenocarcinoma), TGFβ1 has almost no effect on cell growth, but induces a remarkable EMT phenotype (28). Here, we used the TGFβ1-induced EMT in A549 cells to investigate the role of SHP2 in EMT. We found that SHP2 promoted EMT requiring its protein phosphatase activity. The novel interacting protein Hook1, interacting with SHP2 N-terminal SH2 and PTP domains, negatively regulated EMT.

## EXPERIMENTAL PROCEDURES

### 

#### 

##### Cell Culture

A549 and HEK293T cells were cultured in RPMI 1640 and DMEM, respectively (Hyclone, Logan, UT), supplemented with 10% FBS (Hyclone), penicillin (100 units/ml), and streptomycin (100 mg/ml) (Hyclone).

##### Materials

Recombinant human TGFβ1 (mammalian) was from PeproTech (Rocky Hill, NJ). Phenylhydrazonopyrazolone sulfonate 1 (PHPS1) was from Sigma-Aldrich. HOOK1 siRNA (target sequence: GUUGAGAUAUAUCGUCAGA) was from Thermo Fisher Scientific (Dharmacon Products, Lafayette, CO). SHP2 siRNA and all siRNA negative controls were from Santa Cruz Biotechnology Inc. (Santa Cruz, CA). Antibodies for E-cadherin, vimentin, SHP2 and Hook1 were from Santa Cruz Biotechnology. Antibodies for pERK, ERK, pSmad2 (Ser467), pSmad3 (Ser423/425), Smad2, Smad3, COL1A1, and Snail1 were from Cell Signaling Technologies (Beverly, MA). Anti-Myc monoclonal antibody was from OriGene (Rockville, MD). Anti-Flag® antibody produced in rabbit was from Sigma-Aldrich. IRDye 680LT/IRDye 800CW secondary antibodies were from LI-COR Biosciences (Lincoln, NE). Primer synthesis and DNA sequencing were performed by Invitrogen.

##### Plasmids

The Hook1 (NM_015888) human cDNA clone (cat. no. SC114574) was from OriGene (Rockville, MD). The plasmids were generated in the PXJ40 vector as previously described (29). The plasmids used in the yeast two-hybrid system were constructed according to the Clontech protocol (TaKaRa Inc., Otsu, Japan). The mutant plasmids were constructed with the Fast Mutagenesis System (Transgen Biotech, Beijing, China) according to the manufacturer's instructions. Plasmids: pXJ40-FLAG-Hook1 (full-length), -Hook1C (AA573–728), pXJ40-MYC-SHP2 (full-length), -NSH2 (AA2–109), -CSH2 (AA110–220), -PTP (AA240–525), pXJ40-MYC-SHP2 mutant (C459S, E76V); pGADT7-SHP2-PTP (AA240–525), pGADT7-Hook1N (AA2–353), -Hook1M (AA353–573), -Hook1C (AA573–728), pGBKT7-SHP2 (full-length), -NSH2 (AA2–109), -CSH2 (AA110–220), -SHP2-PTP (AA240–525). All primers used for construction are listed in supplemental Table S1.

##### Transwell Migration Assay

For cell migration assays, 1 × 10^5^ A549 cells were plated on 8-μl transwell filters (Corning, Corning, NY). The cells were induced to migrate toward medium containing 10% FBS with or without TGFβ1 (5 ng/ml) for 16 h in a 5% CO_2_ incubator. Non-invading cells were removed with a swab. The remaining cells were fixed in 4% paraformaldehyde, stained with crystal violet, and analyzed under a bright-field microscope. The number of migrating cells that had infiltrated the filter was measured by counting 3 random fields per filter. Mean values were obtained from at least three separate experiments.

##### Protein Extraction and Western Blot Analysis

Protein extracts from cultured cells were prepared in RIPA buffer (50 mm Tris, pH 7.4, 150 mm NaCl, 1% Nonidet P-40, 0.5% sodium deoxycholate, 0.1% SDS) according to the instructions (Beyotime, Beijing, China). Protein concentrations were measured with a protein assay kit (Bio-Rad). Routine Western blot analysis was performed. In brief, cell lysates were separated by SDS-PAGE on 10% or 14% polyacrylamide gels and transferred to a nitrocellulose membrane (Pall, Port Washington, NY). Then, the membrane was blocked with 5% dry milk in TBST (50 mm Tris, 150 mm NaCl, 0.05% Tween 20, pH 7.6) for 1 h at room temperature. The membrane was then incubated with primary antibodies at 4 °C overnight. Afterward, the membrane was washed with TBST and probed with IRDye 680LT/IRDye 800CW secondary antibodies (LI-COR Biosciences, Lincoln, NE) for 1 h at room temperature. Signals were visualized on an Odyssey two-color infrared imaging system (LI-COR Biosciences, Lincoln, NE).

##### Immunoprecipitation

The cells in 100-mm dishes were lysed in 1 ml of RIPA buffer (Beyotime, Beijing, China). The lysate was pre-cleaned with protein A/G-Sepharose beads (Santa Cruz Biotechnology) by rotation at 4 °C for 1 h. After centrifugation, the supernatant was incubated with antibody for 1 h at 4 °C. Then, protein A/G-Sepharose (Santa Cruz Biotechnology) was added and incubated at 4 °C overnight with rotation. Beads were centrifuged and washed three times with RIPA buffer. Then, the beads were suspended with 2× Laemmli sample buffer (Bio-Rad). After boiling for 5 min, proteins were separated by SDS-PAGE, and Western blotting was performed as described above.

##### Real-time PCR Analysis

Total RNA was isolated from cells using TRIzol reagent (Invitrogen) according to the manufacturer's instructions. RNA was reversed to cDNA using the ReverTra Ace qPCR RT Kit (Toyobo Inc., Osaka, Japan). Real-time PCR was performed on a Light-Cycler Roche480 (Roche Molecular Systems) using the Light-Cycler Roche480 master kit. All real-time PCR assays were performed in duplicate, and data were from at least three independent experiments. All values are shown as ratios to *gapdh* levels. The primers used are listed in supplemental Table S2.

##### Yeast Two-hybrid Assay

The yeast two-hybrid screen was performed using the Matchmaker Gold Yeast Two-hybrid System (TaKaRa Inc., Otsu, Japan) and followed the manufacturer's instructions. The screening library was a Mate and Plate library human universal normalized cDNA library (TaKaRa Inc., Otsu, Japan). The bait was constructed by ligating full-length SHP2 or an SHP2-PTP fragment (AA240-525, NM 002834.3) into the BamH I and EcoR I sites of the pGBKT7 vector (TaKaRa Inc., Otsu, Japan). The screen was performed under high-stringency growth conditions as recommended by the manufacturer. Transformations were performed according to the Clontech protocol (TaKaRa Inc., Otsu, Japan).

##### Monolayer Wound-healing Assay

Cells were grown to 100% confluence, wounded by scratching with a pipette tip, and incubated for another 40 h. Phase-contrast images were captured at 0, 16, and 40 h.

##### SHP2 PTP Activity Assay

Cells were harvested in PTP lysis buffer (25 mm Hepes, pH7.4, 150 mm NaCl, 1 mm DTT, 2 mm EDTA, 0.5% Triton X-100) supplemented with protease inhibitor mixture. The supernatants (0.3 mg each) were incubated with anti-SHP2 antibody for 1 h, then protein A/G-Sepharose was added and incubated for 3 h at 4 °C. The immuno-precipitates were washed in the PTP lysis buffer twice and then washed twice with phosphatase reaction buffer (25 mm Hepes, pH7.4, 50 mm NaCl, 1 mm DTT, 0.05% Triton X-100). The immunocomplexes were resuspended in 250 μl of reaction buffer with 50 μm 6,8 difluoro-4-methylumbelliferyl phosphate (DiFUMP) (Invitrogen) and incubated at 37 °C for 30 min. DiFUMP fluorescence signal was measured at an excitation of 355 nm and an emission of 460 nm using SpectraMax M5 plate reader (Molecular Devices, Sunnyvale, CA). Levels of immunoprecipitated SHP2 were analyzed by Western blotting. All values were normalized to the value of control.

##### SBE4-Luc Activity Assay

SBE4-Luc was from Addgene, and pRL-TK was from Promega Corporation (Madison, WI). A549 cells were cotransfected with SBE4-Luc, pRL-TK and control siRNA/SHP2 siRNA. Luciferase activity was detected according to the protocols for Dual-Luciferase Reporter Assay System (Promega) and measured by SpectraMax M5 plate reader (Molecular Devices, Sunnyvale, CA).

##### Statistical Analysis

Quantitative data are expressed as mean ± S.E. Statistical significance was determined by Student's *t* test. *p* value of <0.05 was considered to be statistically significant.

## RESULTS

### 

#### 

##### SHP2 Was Required for EMT

**FIGURE 1.**
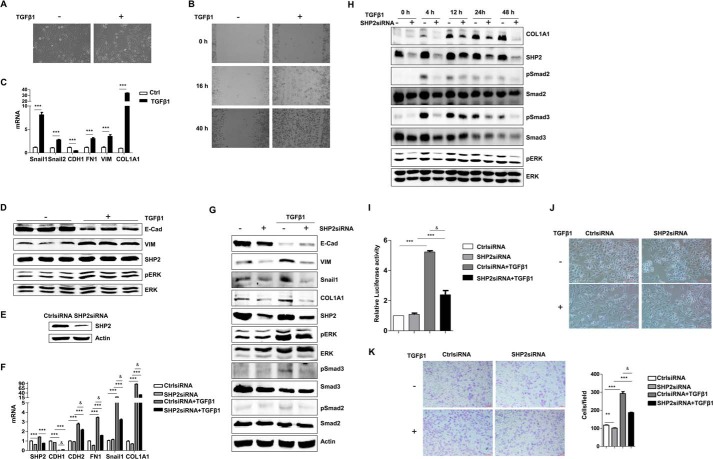
**SHP2 positively regulated EMT.** *A*, morphology of A549 cells with or without TGFβ1 treatment (5 ng/ml) for 24 h. *B*, cell wound assay was used to test the migration of A549 cells, with or without TGFβ1 treatment (5 ng/ml). Images were captured at 0, 16, and 40 h after wounding. *C*, relative mRNA levels of EMT-related transcription factors (*Snail1* and *Snail2*) and markers (*CDH1, FN1, VIM, COL1A1*) detected by real-time PCR in A549 cells with or without TGFβ1 treatment (5 ng/ml) for 24 h (***, *p* < 0.005). *D*, Western blot analysis of the changes in EMT markers (E-Cad, VIM) and the activation of ERK in A549 cells with or without TGFβ1 treatment (5 ng/ml) for 48 h. The assay was performed in three independent experiments under the same conditions. *E*, SHP2 in A549 cells was successfully reduced after SHP2siRNA transfection. *F*, quantification PCR of EMT-related markers in control siRNA (CtrlsiRNA) and SHP2siRNA-transfected A549 cells, with or without TGFβ1 treatment (5 ng/ml) for 24 h. ***, *p* < 0.005 between with and without TGFβ1; &, *p* < 0.005 between CtrlsiRNA and SHP2siRNA. *G*, Western blot analysis of the EMT markers (E-Cad, VIM, Snail, COL1A1) and the activation of ERK, Smad2, Smad3 in control siRNA and SHP2siRNA-transfected A549 cells, with or without TGFβ1 treatment (5 ng/ml) for 48 h. *H*, Western blot analysis of the activation of ERK, Smad2, and Smad3 in SHP2siRNA-transfected A549 cells, with TGFβ1 treatment (5 ng/ml) for 0, 4, 12, 24, 48 h. *I*, SBE4-Luc activity assay to detect the activation of Smad pathway in SBE4-Luc, pRL-TK, control siRNA or SHP2siRNA-transfected A549 cells, with or without TGFβ1 treatment (5 ng/ml) for 8 h. ***, *p* < 0.005 between with and without TGFβ1 treatment; &, *p* < 0.005 between CtrlsiRNA and SHP2siRNA. *J*, morphological changes of control siRNA and SHP2siRNA-transfected A549 cells, with or without TGFβ1 treatment (5 ng/ml) for 24 h. Scale bar, 50 μm. *K*, transwell assays to assess the migration of control siRNA and SHP2siRNA-transfected A549 cells, with or without TGFβ1 treatment (5 ng/ml) for 24 h. Scale bar, 100 μm. ***, *p* < 0.005 between with and without TGFβ1 treatment; &, *p* < 0.005 between CtrlsiRNA and SHP2siRNA.

Non-small cell lung cancer cells (A549) were stimulated with TGFβ1 (5 ng/ml) for 24 h. The cells had notable morphological changes, from slab-like to an elongated spindle shape (Fig. 1*A*). The migration capacity was enhanced as shown by the wound-healing assay (Fig. 1*B*). Quantitative PCR demonstrated decreased transcription of the epithelial gene E-cadherin (*CDH1*) and increased mRNA levels of vimentin (*VIM*), Snail1, Snail2, fibronectin1 (*FN1*), and collagen1 A1 (*COL1A1*) (Fig. 1*C*), demonstrating that the TGFβ1-treated A549 cells lost their epithelial characteristics and gained mesenchymal phenotype.

In the process of EMT in A549 cells induced by TGFβ1, the expression of SHP2 did not change, the activation of ERK pathway was dramatically induced after TGFβ1 stimulation for 48 h (Fig. 1*D*). Then we investigated the effect of knockdown of SHP2 by siRNA on the EMT. SHP2 was successfully decreased by transfection with siRNA (Fig. 1, *E* and *F*). TGFβ1 greatly decreased the mRNA level of the epithelial gene *CDH1*, and this was partially blocked by the decreased expression of SHP2. Accordingly, down-regulation of SHP2 repressed the mRNA levels of mesenchymal genes (*CDH2*, *FN1*, *Snail1*, and *Col1A1*) (Fig. 1*F*). We further confirmed these results by Western blotting (Fig. 1*G*). The increased protein levels of VIM, Snail1, COL1A1 and activation of ERK, Smad pathway were partially attenuated by transfection with SHP2siRNA. We also detected the activation of ERK and Smad pathways at different time points (0, 4, 12, 24, 48 h) with TGFβ1 stimulation when SHP2 was or was not knockdown. The results showed that SHP2 knockdown inhibited TGFβ1 induced Smad and ERK pathway (Fig. 1*H*). TGFβ1 non-Smad pathway-ERK pathway might be important and essential for EMT in A549 cells. Furthermore, we used SBE4-Luc reporter system to test the activation of Smad pathway with or without SHP2 siRNA transfection (30). The activation of Smad pathway was repressed when SHP2 was knockdown (Fig. 1*I*). And the TGFβ1-induced alterations of cell morphology and cell motility were also interrupted by down-regulation of SHP2 (Fig. 1, *J* and *K*). Therefore, SHP2 positively regulated the transition of epithelial to mesenchymal characteristics of A549 cells.

##### Phosphatase Activity of SHP2 Was Required for Regulating EMT

**FIGURE 2.**
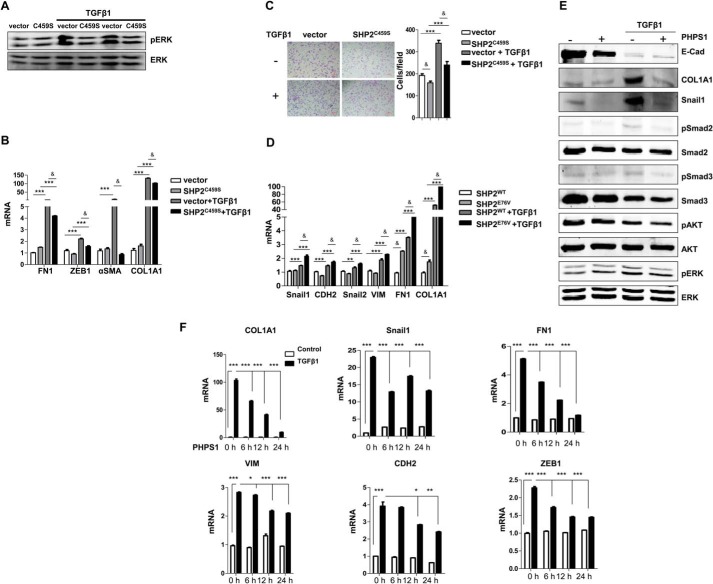
**Phosphatase activity of SHP2 was required for EMT.** *A*, Western analysis of effects of SHP2 C459S overexpression on ERK activation with or without TGFβ1 treatment (5 ng/ml) for 48 h. *B*, quantification of EMT-associated markers (*FN1, ZEB1*, α*SMA, COL1A1*) in empty vector and SHP2^C459S^-transfected A549 cells, with or without TGFβ1 treatment (5 ng/ml) for 24 h. ***, *p* < 0.005 between with and without TGFβ1 treatment; &, *p* < 0.01 between vector and SHP2^C459S^. *C*, transwell assay was used to detect the migration of empty vector and SHP2^C459S^-transfected A549 cells, with or without TGFβ1 treatment (5 ng/ml) for 16 h. Scale bar, 100 μm. ***, *p* < 0.005 between with and without TGFβ1 treatment; &, *p* < 0.01 between vector and SHP2^C459S^. *D*, QPCR analysis of EMT-related markers (*Snail1, Snail2, CDH2, VIM, FN1, COL1A1*) in A549 cells transfected with SHP2^WT^ and SHP2^E76V^, with or without TGFβ1 treatment (5 ng/ml) for 24 h. ***, *p* < 0.005 between with and without TGFβ1 treatment; &, *p* < 0.05 between SHP2WT and SHP2^E76V^. *E*, Western blot analysis of changes in EMT markers (E-Cad, COL1A1, Snail1) and the activation of ERK, Smad2, Smad3 in control and PHPS1 (20 μm)-treated A549 cells, with or without TGFβ1 treatment (5 ng/ml) for 48 h. *F*, QPCR analysis of EMT-related markers (*COL1A1, Snail1, FN1, VIM, CDH2, ZEB1*) in A549 cells treated with PHPS1 (20 μm) for 0, 6, 12, and 24 h in the absence or presence of TGFβ1 (5 ng/ml) for 24 h. (*, *p* < 0.05; **, *p* < 0.01; ***, *p* < 0.005).

To further investigate whether the phosphatase activity of SHP2 is involved in EMT, the phosphatase active center of SHP2 was mutated by substitution of Cys-459 to Ser. This phosphatase-dead mutation (SHP2^C459S^) markedly repressed the TGFβ1-induced ERK activation in A549 cells (Fig. 2*A*). Similar to SHP2 siRNA, the SHP2^C459S^ mutation also partially blocked the TGFβ1-induced EMT in A549 cells as indicated by repressed mRNA levels of transcriptor and mesenchymal genes (*ZEB1*, *FN1*, α*SMA*, and *Col1A1*) (Fig. 2*B*) and cell migration (Fig. 2*C*). Another mutation, E76V, that releases SHP2 from auto-inhibition, was also used. The results demonstrated that the constitutively activated SHP2 mutant (SHP2^E76V^) promoted the mesenchymal phenotype in A549 cells as indicated by increased mRNA levels of transcriptor and mesenchymal genes (*Snail1*, *Snail2*, *CDH2*, *FN1*, *VIM*, and *COL1A1*) (Fig. 2*D*). Overexpression of wild-type SHP2 also slightly enhanced the mesenchymal gene transcription. To further confirm that phosphatase enzymatic activity was required for its role in promoting EMT, SHP2 inhibitor PHPS1 was introduced. We examined the effect of PHPS1 on the protein levels of EMT-associated markers. The results showed that the increased levels of Snail1, COL1A1 and activation of ERK and Smad pathways with TGFβ1 stimulation were interrupted by the PHPS1 treatment (Fig. 2*E*). Our results showed that PHPS1 attenuated the EMT phenotype in a time-dependent manner (Fig. 2*F*). Our data strongly suggested that SHP2 was positively involved in the process of TGFβ1-induced EMT and its enzymatic activity was required.

##### Hook1 Interacted with SHP2

**FIGURE 3.**
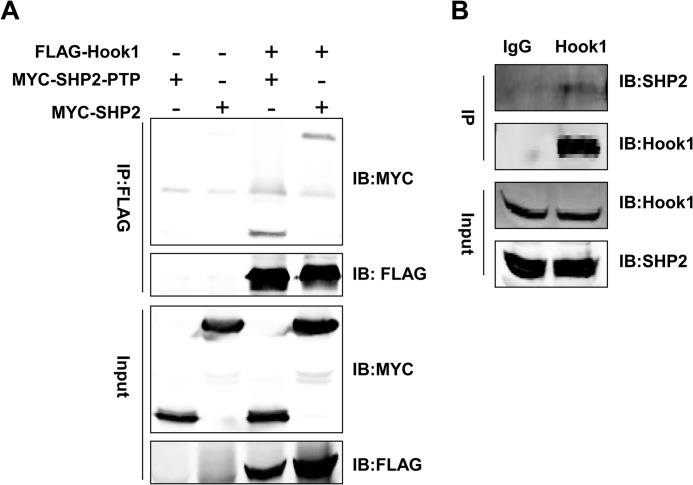
**Hook1 was a new binding protein of SHP2.** *A*, co-immunoprecipitation assay to verify the interaction of overexpressed full-length SHP2 (MYC-SHP2) or PTP domain of SHP2 (MYC-SHP2-PTP) with FLAG-Hook1 in HEK293T cells. *B*, co-immunoprecipitation assay to verify the endogenous interaction of SHP2 with Hook1 in HEK293T cells.

To discover the molecular mechanisms that by which SHP2 promotes EMT and how SHP2 is activated in EMT, yeast two-hybrid assays were performed. Both full-length and the protein-tyrosine phosphatase domain of SHP2 were constructed in pGBKT7 as baits. When full-length SHP2 was used, there were no positive clones. When the PTP domain of SHP2 was used, there were several positive clones (supplemental Fig. 1*A*). DNA sequencing showed that Hook1 was one of the positive clones with the correct reading frame (supplemental Fig. 1*B*). To further confirm the interaction, either full-length SHP2 or its PTP was co-transformed with plasmid containing Hook1 into yeast cells, the results showed that SHP2-PTP but not full-length SHP2 interacted with Hook1 (supplemental Fig. 1, *C* and *D*). Further, co-immuno-precipitation assay in HEK293T cells showed that both MYC-tagged SHP2 and its PTP domain interacted with FLAG-tagged Hook1 (Fig. 3*A*). We further showed that endogenous SHP2 was co-immunoprecipitated with Hook1 from the lysate of HEK293T cells (Fig. 3*B*). We thus demonstrated that Hook1, an endogenous SHP2-interacting protein, bound to its PTP domain.

##### Hook1 Negatively Regulated EMT

**FIGURE 4.**
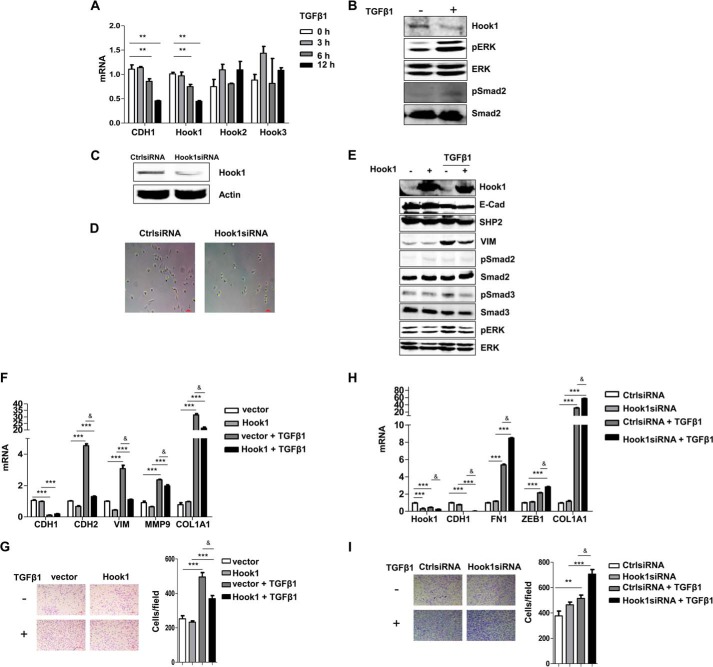
**Hook1 negatively regulated EMT.** *A*, quantification of the mRNA levels of *CDH1*, *Hook1, Hook2*, and *Hook3* in A549 cells with TGFβ1 treatment (5 ng/ml) for 0, 3, 6, and 12 h (**, *p* < 0.01). *B*, Western blot analysis of the expression levels of Hook1 after treatment with TGFβ1 (5 ng/ml) for 48 h. *C*, Hook1 was knockdown after Hook1siRNA transfection in A549 cells. *D*, cell adhesion in control siRNA and Hook1siRNA-treated A549 cells. *E*, Western blot analysis of the effects of Hook1 overexpression on EMT markers (E-Cad and VIM) and ERK, Smad2, Smad3 activation in A549 cells with or without TGFβ1 treatment (5 ng/ml) for 24 h. *F*, QPCR analysis of EMT markers (*CDH1*, *CDH2*, *VIM*, *MMP9*, and *COL1A1*) under the same conditions as *E*. ***, *p* < 0.005 between with and without TGFβ1 treatment; &, *p* < 0.01 between vector and Hook1. *G*, transwell assays to assess the migration of control and Hook1-transfected A549 cells, with or without TGFβ1 treatment (5 ng/ml) for 16 h. Scale bar, 100 μm. ***, *p* < 0.005 between with and without TGFβ1 treatment; &, *p* < 0.01 between vector and Hook1. *H*, QPCR analysis of EMT-related markers (*CDH1*, *FN1*, *ZEB1*, and *COL1A1*) in control siRNA and Hook1siRNA-transfected A549 cells, with or without TGFβ1 treatment (5 ng/ml) for 24 h. ***, *p* < 0.005 between with and without TGFβ1 treatment; &, *p* < 0.05 between CtrlsiRNA and Hook1siRNA. I, transwell assays to assess the migration of control and Hook1siRNA-treated A549 cells, with or without TGFβ1 treatment (5 ng/ml) for 16 h. Scale bar, 100 μm. ***, *p* < 0.005 between with and without TGFβ1 treatment; &, *p* < 0.05 between CtrlsiRNA and Hook1siRNA.

It has been reported that Hook1 is a microtubule-binding protein and participates in cell skeleton reorganization. To explore the possibility that Hook1 plays a role in EMT, we first found that Hook1 was down-regulated at both the mRNA and protein levels in TGFβ1-stimulated A549 cells (Fig. 4, *A* and *B*). Three members of the Hook family have been reported (31–33). Expression of the other two members, Hook2 and Hook3, did not change in the process of TGFβ1 induced-EMT (Fig. 4*A*). Knockdown of Hook1 reduced cell adhesion (Fig. 4, *C* and *D*). Overexpression of Hook1 inhibited the activation of ERK induced by TGFβ1 as well as the EMT phenotype indicated by the blockade of TGFβ1-induced expression of VIM (Fig. 4*E*). This was further confirmed by the transcriptional levels of the epithelial gene *CDH1* and mesenchymal genes (*CDH2*, *VIM*, *MMP9*, and *COL1A1*) (Fig. 4*F*). Cell migration was also repressed by overexpression of Hook1 (Fig. 4*G*). In line with these observations, knockdown of Hook1 by siRNA promoted the expression of mesenchymal genes and cell migration (Fig. 4, *H* and *I*). These results indicated that Hook1 was a negative regulator of EMT.

##### Implications of Hook1 and SHP2 Interaction in EMT

**FIGURE 5.**
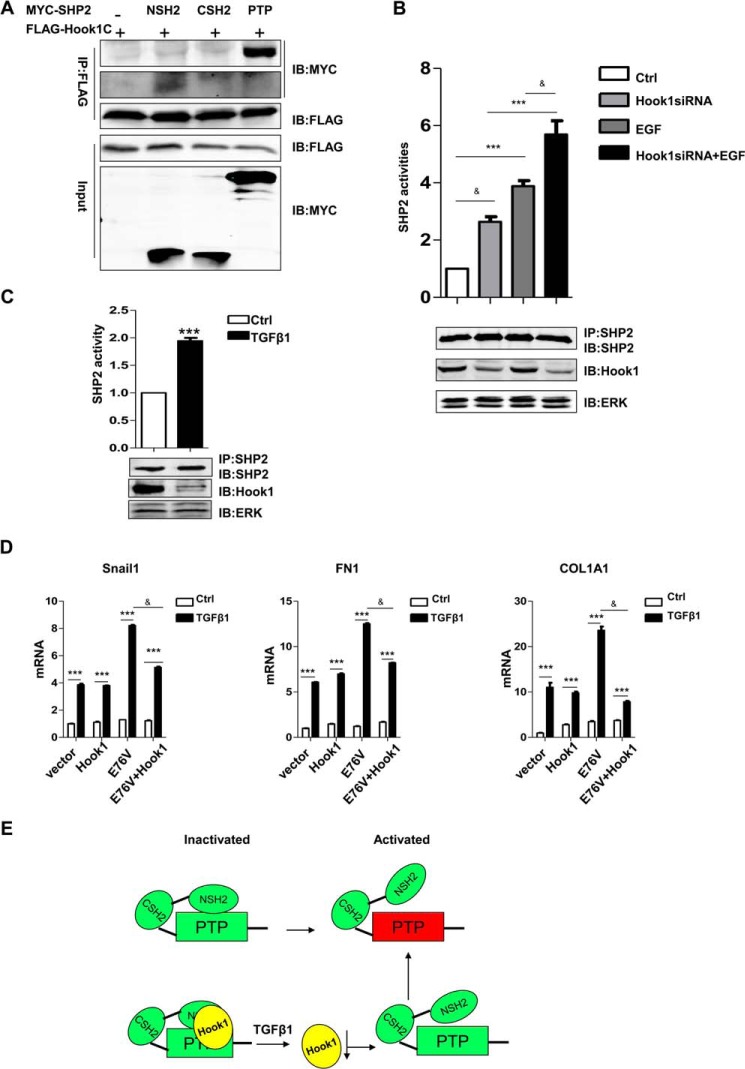
**Interaction of SHP2-NSH2 and PTP domains with Hook1.** *A*, co-immunoprecipitation assay to verify the direct interaction of FLAG-Hook1C with either MYC-SHP2-NSH2 or MYC-SHP2-PTP in HEK293T cells. *B*, PTP activity assay to test SHP2 activity in immunocomplex from control siRNA or Hook1siRNA-transfected HEK293T cells, with or without EGF (50 ng/ml) stimulation for 15 min. ***, *p* < 0.005 between Ctrl and EGF; &, *p* < 0.05 between CtrlsiRNA and Hook1siRNA. *C,* PTP activity assay to test SHP2 activity in immunocomplex from A549 cells with or without TGFβ1 (5 ng/ml) stimulation for 24 h (***, *p* < 0.005). *D*, QPCR analysis of mRNA levels of EMT markers (*Snail1*, *COL1A1*, *FN1*) in vector, Hook1, SHP2^E76V^-transfected, Hook1 and E76V co-transfected A549 cells, with or without TGFβ1 treatment (5 ng/ml) for 24 h. ***, *p* < 0.005 between with and without TGFβ1 treatment; &, *p* < 0.05 between SHP2E76V only and SHP2E76V plus Hook1. *E*, schematic model of the interaction between SHP2 and Hook1 in EMT. Hook1 locks the auto-inhibition caused by the intramolecular interaction between N-SH2 and PTP. Upon TGFβ1 treatment, Hook1 is reduced, and this weakens the interaction between N-SH2 and PTP, leading to the full activation of SHP2 by other molecules, including substrates.

The results showed that both SHP2 and Hook1 were involved in EMT but acted reversely. Therefore, we investigated the interaction between SHP2 and Hook1 in more detail. We found that the C terminus of Hook1 (Hook1-C) bound not only to the PTP domain of SHP2, similar to the yeast two-hybrid screening results, but also to the N terminus SH2 domain (N-SH2) (supplemental Table S3 and Fig. 1, *E* and *F*). Next, the interactions were verified by *in vitro* co-immuno-precipitation experiment (Fig. 5*A*). Our data demonstrated that both the N-SH2 and PTP domains of SHP2 interacted directly with the Hook1 C-terminal. We further tested SHP2 PTP activity in the SHP2 immuno-complex, and found that when HEK293T cells were transfected with Hook1 siRNA, SHP2 activity was up-regulated with or without EGF stimulation for 15 min (Fig. 5*B*). And further, SHP2 activity was also up-regulated after TGFβ1 stimulation for 24 h in A549 (Fig. 5*C*). Moreover, co-expression of Hook1 and SHP2^E76V^ inhibited the promotional effect of SHP2^E76V^ on EMT (Fig. 5*D*). So we supposed that the direct interactions of Hook1 with SHP2 NSH2 and PTP domains inhibited the activation of SHP2. With TGFβ1 stimulation, the expression of Hook1 was down-regulated, and SHP2 was easy to be activated (Fig. 5*E*).

## DISCUSSION

A widely expressed and important phosphatase, SHP2 plays essential roles in various cell functions, including cell proliferation, metabolism, migration, and transformation (4, 34, 36–39). EMT is a form of transformation, which is important for cancer cells to obtain the capacities of migration and invasion. Our data showed that SHP2 positively regulated TGFβ1-induced EMT in A549 cells (Fig. 1, *F–J*). SHP2 was required for TGFβ1-induced Smad and ERK pathway. This indicated that SHP2 may also participate in other cell function and signal pathways that TGFβ1 regulated, including extracellular matrix synthesis and deposition. Furthermore, the activated mutant SHP2^E76V^ promoted the expression of mesenchymal genes (Fig. 2*D*). Dominant negative mutation of SHP2 or the SHP2 inhibitor PHPS1 partially inhibited the EMT (Fig. 2, *B*, *E*, *F*). It has been reported that inhibition of SHP2 promotes mesenchymal-to-epithelial transformation in breast cancer cells (40), which was consistent with our current finding that SHP2 promoted EMT in lung epithelial cells. Somatic activated SHP2 mutations have also been detected in neuroblastoma, melanoma, acute myeloid leukemia, breast cancer, lung cancer, and colorectal cancer. Our findings strongly suggest that SHP2 is a key regulator in tumor metastasis by promoting EMT.

We have identified a novel interacting protein Hook1, the function of which is opposite to that of SHP2 in the process of EMT. Hook1 is a microtubule-binding protein that participates in spermatogenesis, cell differentiation, and endocytosis (31, 41–45). Hook1 has been mentioned in previous EMT studies (46, 47). The claudin-low and metaplastic subtypes of breast cancer display a significant expression pattern of EMT transcriptional factors, which correlates negatively with a pathological complete response (48). Gene expression extracted from microarray data showed that either overexpression of one of the EMT transcription factors TGFβ, Twist, Gsc, or Snaill or knockdown of E-cadherin significantly down-regulates the expression of Hook1 (48). Hook1 is down-regulated in colon cancer cells by inducible expression of hSnaill (46, 47, 49). Bioinformatics analysis showed that there is an E-BOX motif (CAGGTG) in the promoter region of *Hook1*, which might be recognized by the important EMT-related transcriptors Snaill, Slug, and ZEB1 (46). This supports the idea that *Hook1* is a target gene in EMT. In the A549 cells, with TGFβ1 stimulation, the expression of EMT-related transcriptors was increased, which suppressed the expression of Hook1. Our results provided strong evidence that microtubule-associated Hook1 was negatively associated with EMT. Recently, it was reported that EMT-associated cell skeleton changes were microtubule-dependent, indicating that Hook1, as a microtubule-binding protein, participated the regulation of cytoskeletal reorganization in EMT (50). Confirmation that Hook1 is negatively involved in the process of EMT suggests that it might be a potential target for interrupting EMT in cancer cell, which is important for metastasis.

Hook1 was identified to be a SHP2-interacting protein when the SHP2 PTP domain was used as bait. Further studies showed that both the N-SH2 and PTP domains of SHP2 interacted with the Hook1 C terminus. Interestingly, in the inactive state, SHP2 is auto-inhibited by N-SH2 binding to the PTP domain. SHP2 activation requires detachment of the NSH2 domain from the PTP domain. SHP2 activity was increased by TGF treatment through down-regulation of Hook1. Co-expression of Hook1 and SHP2^E76V^ could partially offset the promotional role of SHP2^E76V^ in TGFβ1-induced EMT. EGF also activated SHP2 without affected the protein levels of Hook1. We proposed that SHP2 enzymatic activity is temporally and spatially regulated through scaffolding proteins, including Hook1. It is likely that SHP2 is activated rapidly by EGF stimulation, generating a faster response without affecting the Hook1 protein level. Alternatively, TGFβ1-induced EMT may require the persistent activation of SHP2 where Hook1 transcriptional regulation is initiated. The expression of Hook1 is reduced by transcription factors such as Snaill and ZEB1, and further loosens the interaction between SHP2-NSH2 and PTP, so SHP2 becomes easier to be activated and promotes EMT (35, 51–53). These results suggested that Hook1 was a negative regulator of SHP2 activation (Fig. 5*E*). In line with this finding, Hook1, in contrast to SHP2, negatively regulated the TGFβ1-induced EMT.

## Supplementary Material

Supplemental Data

## Footnotes

3 The abbreviations used are:

EMT: epithelial-mesenchymal transition
SH2: Src homology 2
SHP: SH2-domain containing protein-tyrosine phosphatase
DiFUMP: 6,8 difluoro-4-methylumbelliferyl phosphate
PHPS: phenylhydrazonopyrazolone sulfonate.
