# Supplementary material for: SHP2 Positively Regulates TGFβ1-induced Epithelial-Mesenchymal Transition Modulated by Its Novel Interacting Protein Hook1
Source: J Biol Chem. 2014 Oct 20;289(49):34152–60. doi: 10.1074/jbc.M113.546077 (PMC4256348; doi:10.1074/jbc.M113.546077)
Supplement: Supplemental Data [file supp_289_49_34152__index.html]

SHP2 Positively Regulates TGFβ1-induced Epithelial-Mesenchymal Transition Modulated by Its Novel Interacting Protein Hook1 — SHP2-Hook1 Complex Regulates TGFβ1-induced EMT — Supplemental Data 

# SHP2 Positively Regulates TGFβ1-induced Epithelial-Mesenchymal Transition Modulated by Its Novel Interacting Protein Hook1

## Supplemental Data

**Files in this Data Supplement:**

- Supplemental data
