# Supplementary material for: SHP2 Positively Regulates TGFβ1-induced Epithelial-Mesenchymal Transition Modulated by Its Novel Interacting Protein Hook1
Source: J Biol Chem. 2014 Oct 20;289(49):34152–60. doi: 10.1074/jbc.M113.546077 (PMC4256348; doi:10.1074/jbc.M113.546077)
Supplement: Supplemental Data [file supp_M113.546077_M546077-zbc0318-SUPPLEMENTAL_DATA.doc]

SHP2 Positively Regulates TGFβ1-induced Epithelial-mesenchymal Transition Modulated by Its Novel Interacting Protein Hook1*

**Shuomin Li^1^, Linrun Wang^2^, Qingwei Zhao^2^, Yu Liu^1^, Lingjuan He^2^, Qinqin Xu^1^, Xu Sun^1^, Li Teng^1^, Hongqiang Cheng^1,3^ and Yuehai Ke^1,3^**

1. Department of Pathology and Pathophysiology, Program in Molecular Cell Biology, Zhejiang University School of Medicine, Hangzhou 310058, China
2. The First Affiliated Hospital, College of Medicine, Zhejiang University, Hangzhou 310003, China
3. Collaborative Innovation Center for Diagnosis and Treatment of Infectious Diseases, Hangzhou, Zhejiang 310003, China

***Running title: *SHP2-Hook1 complex regulates TGFβ1-induced EMT*

*To whom correspondence should be addressed: Yuehai Ke, Ph.D. (Email: yke@zju.edu.cn; Tel: 86-571-88208713; Fax: 86-571-88208583) and Hongqiang Cheng, Ph.D. (Email: hqcheng11@zju.edu.cn; Tel: 86-571-88981476), Department of Pathology and Pathophysiology, Program in Molecular Cell Biology, Zhejiang University School of Medicine, 866 Yuhangtang Road, Hangzhou 310058, China.

**Keywords:** SHP2, Hook1, epithelial-mesenchymal transition**,** protein tyrosine phosphatase

**SUPPLEMENTAL DATA**

**Table S1. Primers used for construction plasmids**

| **Primer** | **Sequence(5’→3’)** |
| --- | --- |
| pXJ40-MYC-SHP2E76VF | TTGCCACTTTGGCTGTGTTGGTCCAG |
| pXJ40-MYC-SHP2E76VR | ACAGCCAAAGTGGCAAATTTCTCCC |
| pXJ40-MYC-SHP2C459SF | CCGGTCGTGGTGCACTCCAGTGCTGGA |
| pXJ40-MYC-SHP2C459SR | GAGTGCACCACGACCGGCCCTGCATCCA |
| pXJ40-MYC-SHP2F | CGCGGATCCACATCGCGGAGATGGTTTCACCCA |
| pXJ40-MYC-SHP2R | CGGGGTACCTCATCTGAAACTTTTCTGCTGTTGCATCAGGC |
| pXJ40-MYC-SHP2PTPF | CGCGGATCCACAGATAAAGTCAAACAAGGCTTTTGGGA |
| pXJ40-MYC-SHP2PTPR | CGGGGTACCCTATAGTGTTTCAATATAATGCTGGACCGCC |
| pXJ40-MYC-SHP2NF | CGCGGATCCACATCGCGGAGATGGTTTCACCCAAATATC |
| pXJ40-MYC-SHP2NR | CGGGGTACCCTAAGAGGTAGGATCTGCACAGTTCAGAG |
| pXJ40-MYC-SHP2CF | CGCGGATCCGAAAGGTGGTTTCATGGACATCTCTCTG |
| pXJ40-MYC-SHP2CR | CGGGGTACCCTAACGAGTCGTGTTAAGGGGCTGCTTG |
| pXJ40-FLAG-Hook1F | CCGCTCGAGGAGGAGACGCAGCCGCCGCCGCA |
| pXJ40-FLAG-Hook1R | CGGGGTACCTTAATCAGATGTTGTAGCAGGGACTTTAACA |
| pXJ40-FLAG-Hook1CF | CCGCTCGAGCTTCAGCCAGATATAAATCAAAATG |
| pXJ40-FLAG-Hook1CR | CGGGGTACCTTAATCAGATGTTGTAGCAGGGAC |
| pGADT7-Hook1NF | CGCGGATCCATGAGGAGACGCAGCCGCCGCCGCAGCC |
| pGADT7-Hook1NR | CCGCTCGAGCATTATGCATATACATCATGTTGG |
| pGADT7-Hook1MF | CGCGGATCCATAATACAGTCAGCTTAGAAGAAG |
| pGADT7-Hook1MR | CCGCTCGAGCATCTTCAATGAGTTCTTGTTTC |
| pGADT7-SHP2PTPF | CCGGAATTCACAGATAAAGTCAAACAAGGCTTTTGGGA |
| pGADT7-SHP2PTPR | CGGGATCCCTATAGTGTTTCAATATAATGCTGGACCGCC |
| pGBKT7-SHP2FLF | CCGGAATTCACATCGCGGAGATGGTTTCACCCA |
| pGBKT7-SHP2FLR | CGGGATCCTCATCTGAAACTTTTCTGCTGTTGCATCAGGC |
| pGBKT7-SHP2PTPF | CCGGAATTCACAGATAAAGTCAAACAAGGCTTTTGGGA |
| pGBKT7-SHP2PTPR | CGGGATCCCTATAGTGTTTCAATATAATGCTGGACCGCC |
| pGBKT7-SHP2NSH2F | CCGGAATTCACATCGCGGAGATGGTTTCAC |
| pGBKT7-SHP2NSH2R | CGGGATCCCTAAGAGGTAGGATCTGCACAGTTCAGAG |
| pGBKT7-SHP2CSH2F | CCGGAATTCGAAAGGTGGTTTCATGGACATCTCTCTG |
| pGBKT7-SHP2CSH2R | CGGGATCCCTAACGAGTCGTGTTAAGGGGCTG |

Plasmids: pXJ40-FLAG-Hook1(Full length), -Hook1C(AA573-728), pXJ40-MYC-SHP2 (Full length), -NSH2(AA2-109), -CSH2(AA110-220), -PTP(AA240-525); pXJ40-MYC-SHP2-mutant(C459S,E76V), pGADT7-SHP2-PTP(AA240-525), pGADT7-Hook1N(AA2-353), -Hook1M(AA353-573), pGBKT7-SHP2(Full length), -NSH2(AA2-109), -CSH2(AA110-220), -SHP2-PTP(AA240-525).

**Table S2. Primers used for RT-PCR analysis**

| **Primer** | **Sequence(5’→3’)** |
| --- | --- |
| Hook2F | AGCTTCATGAGGCAGATCTGGAGT |
| Hook2R | TCCGCGTCCTTCTTCTGCAAGTTA |
| Hook3F | AAGGCAGCTGAAGATAGACTGGCA |
| Hook3R | TTGAGCGGCACAACTTCTCTACCT |
| MMP9F | TGTACCGCTATGGTTACACTCG |
| MMP9R | GGCAGGGACAGTTGCTTCT |
| Hook1F | TGCTGCTGAGATTATGCCAGTGGA |
| Hook1R | TCAGCCTCTGCTCAGTTTCCAGTT |
| FN1F | GGCTGAAGACACAAGGAAATAAG |
| FN1R | CATTTGAGTTGCCACCGTAAG |
| SHP2F | AAAGGGGAGAGCAATGACGG |
| SHP2R | CTCCACCAACGTCGTATTTCA |
| GAPDHF | GCCTCAAGATCATCAGCAATGCCT |
| GAPDHR | TGTGGTCATGAGTCCTTCCACGAT |
| VIMF | AGAACCTGCAGGAGGCAGAAGAAT |
| VIMR | TTCCATTTCACGCATCTGGCGTTC |
| CDH2F | TGTGGGAATCCGACGAATGGATGA |
| CDH2R | TGGAGCCACTGCCTTCATAGTCAA |
| CDH1F | TGGGCCAGGAAATCACATCCTACA |
| CDH1R | TTGGCAGTGTCTCTCCAAATCCGA |
| ZEB1F | ATACCTGTGAATGGGCGACCAAGA |
| ZEB1R | ACTGCCTGGTGATGCTGAAAGAGA |
| Snail2F | ACCTTGTGTTTGCAAGATCTGCGG |
| Snail2R | TGCAAATGCTCTGTTGCAGTGAGG |
| Snail1F | ACTGCAACAAGGAATACCTCAG |
| Snail1R | GCACTGGTACTTCTTGACATCTG |
| ASMAF | ACCCGCCCAGAAACTAGACACAAT |
| ASMAR  COL1A1F  COL1A1R | TCGCCCACGTAGGAATCTTTCTGA  GAGGGCCAAGACGAAGACATC  CAGATCACGTCATCGCACAAC |

**Table S3. Results of yeast co-transformed on QDO/X/A plates.**

| **Hook1N (AA2-353) Hook1M (AA353-573) Hook1C (AA573-728)** | | | |
| --- | --- | --- | --- |
| **SHP2-NSH2** | **N** | **N** | **Y** |
| **SHP2-PTP** | **N** | **N** | **Y** |
| ***SHP2-CSH2*** | **N** | **N** | **N** |

**Plasmids used: pGADT7-Hook1N, pGADT7-Hook1M, pGADT7-Hook1C, pGBKT7-SHP2-NSH2, pGBKT7-SHP2-PTP, and pGBKT7-SHP2-CSH2. Y, positive for interaction, N, negative for interaction.**


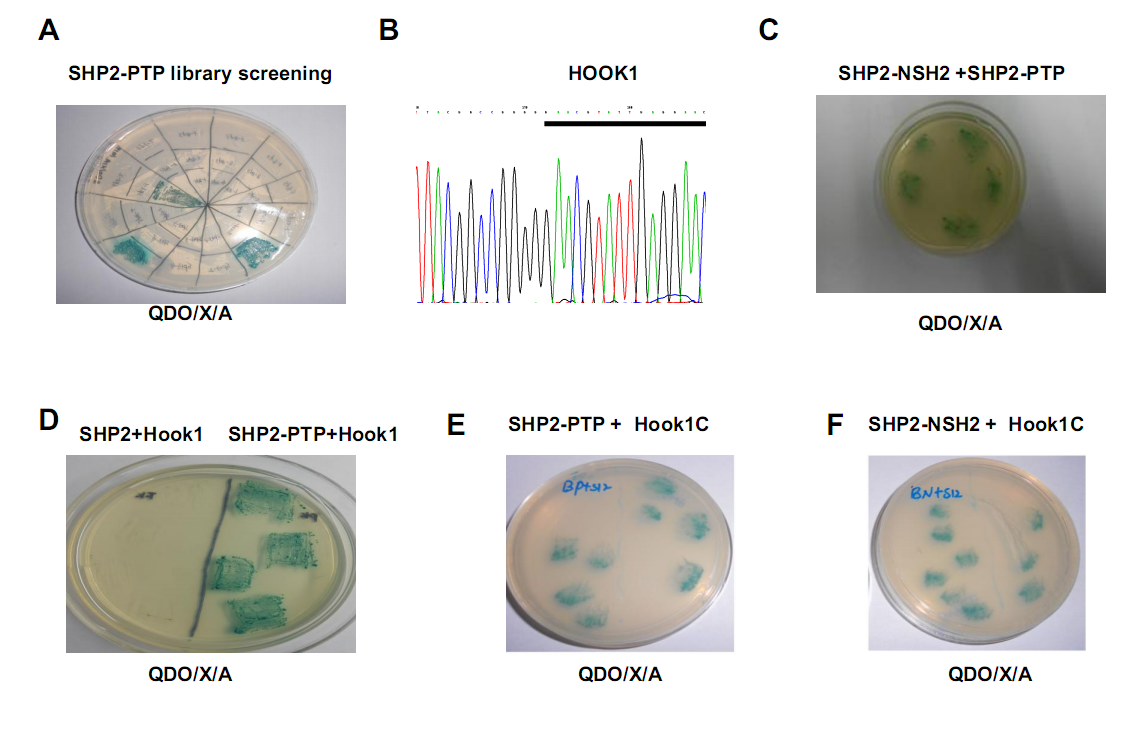


Supplemental Figure*.*SHP2 interacts with Hook1 in yeast two hybrid systems.

*A,* A representative screening result using pGBKT7-SHP2-PTP as bait on SD/-Trp-Leu-Ade-His/X-α-Gal/AbA (QDO/X/A) plates.

*B,* Hook1 was one of the positive clones with the correct reading frame showed by the DNA sequencing.

*C,* The interaction between pGBKT7-SHP2-NSH2 and pGADT7-SHP2-PTP as a positive control.

*D,* The interaction between either pGBKT7-SHP2-PTP or pGBKT7-SHP2 and pGADT7-Hook1.

*E,* The interaction between pGADT7-Hook1C and pGBKT7-SHP2-PTP.

*F,* The interaction between pGADT7-Hook1C and pGBKT7-SHP2-NSH2.
